# New Dental Journals

**Published:** 1874-02

**Authors:** 


*New Dental Journals.*—Two dental journals having recently gone out of existence, the *Dental Times* and *Canada Journal*, their places have been filled by appearance of two new publications named respectively the "*Pennsylvania Journal of Dental Science*," edited and published by Samuel Welchens, D. D. S., Lancaster, Pa., and "*Johnstons' Miscellany*," published in New York by Johnston Brothers.

Dr. Welchens, the editor of the "*Pennsylvania Journal of Dental Science*," is a graduate of the Baltimore College of Dental Surgery, of the class of 1859, since which time he has been in successful practice in Lancaster, Pa., and has occupied a prominent position in the Pennsylvania State Dental Society. The first No. of the new publication contains a photograph and biographical sketch of the late Prof. Chapin A. Harris, A. M., M. D., D. D. S., written by Dr. Jno McCalla, an early graduate of the Baltimore College, and is in every respect a well prepared and interesting number. The "*Dental Miscellany*," from the New York Dental Depot firm of Johnston Brothers, contains among others, two short articles on the "Fractures of the Inferior Maxilla," by N. W. Kingsley, D. D. S., and "Syphilitic Teeth," by T. B. Hitchcock, M. D., D. D. S., and selections from other journals; it presents a very creditable appearance, and promises to be a useful addition to dental magazine literature.

